# Genetic Landscape of Nephropathic Cystinosis in Russian Children

**DOI:** 10.3389/fgene.2022.863157

**Published:** 2022-04-28

**Authors:** K. V. Savostyanov, A. A. Pushkov, O. A. Shchagina, V. V. Maltseva, E. A. Suleymanov, I. S. Zhanin, N. N. Mazanova, A. P. Fisenko, P. S. Mishakova, A. V. Polyakov, E. V. Balanovska, R. A. Zinchenko, A. N. Tsygin

**Affiliations:** ^1^ National Medical Research Center for Children’s Health Federal State Autonomous Institution of the Ministry of Health of the Russian Federation, Moscow, Russia; ^2^ Research Centre for Medical Genetics, Moscow, Russia; ^3^ Ministry of Public Health, Republic of Chechnya, Grozny, Russia

**Keywords:** cystinosis, cystine, children, lysosomal storage diseases, selective screening, therapy monitoring, novel mutations in the CTNS gene

## Abstract

Nephropathic cystinosis is a rare autosomal recessive disorder characterized by amino acid cystine accumulation and caused by biallelic mutations in the *CTNS* gene. The analysis methods are as follows: tandem mass spectrometry to determine the cystine concentration in polymorphonuclear blood leukocytes, Sanger sequencing for the entire coding sequence and flanking intron regions of the *CTNS* gene, multiplex PCR to detect a common mutation—a 57 kb deletion, and multiplex ligation-dependent probe amplification to analyze the number of exon copies in the *CTNS* gene. Haplotype analysis of chromosomes with major mutations was carried out using microsatellite markers D17S831, D17S1798, D17S829, D17S1828, and D17S1876. In this study, we provide clinical, biochemical, and molecular genetic characteristics of 40 Russian patients with mutations in the *CTNS* gene, among whom 30 patients were selected from a high-risk group of 85 people as a result of selective screening, which was carried out through cystine concentration measurement in polymorphonuclear blood leukocytes. The most common pathogenic variant, as in most described studies to date, was the 57 kb deletion, which represented 25% of all affected alleles. Previously non-described variants represented 22.5% of alleles. The founder effect in the Karachay and Chechen ethnic groups was shown for the following major variants: c.1015G > A and c.518A > G.

## Introduction

Nephropathic cystinosis is a rare hereditary disorder caused by the mutations in the *CTNS* gene, which encodes cystinosin, a lysosomal cystine transporter. It contains 367 amino acid residues and transports cystine via lysosomal proton gradient (Ruivo et al., 2012). Biallelic mutations in this gene lead to protein function disruption and intralysosomal cystine crystal accumulation in cells of various organs and tissues, predominantly the kidneys. The pathogenic variants in the *CTNS* gene, which are located at 17р13.2, lead to cystinosin defects, causing disruptions in cystine transportation into the cytoplasm, which leads to gradual intralysosomal cystine accumulation with subsequent crystallization due to its low solubility in water.

The genetic landscape of cystinosis varies greatly depending on the ethnicity (David et al., 2019) and geographic location (Ebbesen et al., 1976; Anikster et al., 1999; Hult et al., 2014; Angileri et al., 2015). The highest incidence (1:3600) was found in Pakistani people in the West Midlands, United Kingdom (Hutchesson et al., 1998). The number of affected people is constantly growing, and 15–20 new cases per year are registered only in the United States (Nesterova and Gahl, 2008).

There are three types of cystinosis based on the symptom severity and age of manifestation: classic infantile nephropathic (OMIM 219800), juvenile nephropathic (OMIM 219900), and adult non-nephropathic (OMIM 219750).

As of date, 161 pathogenic *CTNS* variants are described in the HGMD Professional 2021.2 international database (https://portal.biobase-international.com/hgmd/pro/gene.php?gene=CTNS). The most common variant, according to the literature, is the 57 kb deletion, which partially affects the following genes: *CTNS*, *TRPV1*, and *CARKL*. This deletion is detected in patients with cystinosis in approximately 75% of all the described European cases (Touchman et al., 2000; Kalatzis, 2002). Patients with this deletion are characterized by a distinct extrarenal cystinosis phenotype and early mortality (Gahl et al., 2007).

As of date, the golden standard of laboratory diagnostics for cystinosis is tandem mass spectrometry with high-performance liquid chromatography (HPLC), which is widely used for treatment monitoring due to its high sensitivity and specificity, allowing it to detect the cystine concentrations as low as 0.02 μmol/L (Chabli et al., 2007). The confirmatory diagnostic method is molecular genetic diagnostics directed at pathogenic variants in the *CTNS* gene. The introduction of tandem mass spectrometry and genetic diagnostics in modern clinical practice in recent years allows to successfully detect patients with cystinosis in high-risk groups at an early age and to monitor the pathogenetic therapy (Savostyanov et al., 2018).

We provide clinical, genographic, and molecular genetic data of Russian patients with nephropathic cystinosis in this study.

## Materials and Methods

### Subjects

The examined cohort included 40 children with a clinical diagnosis of “nephropathic cystinosis,” confirmed by molecular genetic methods: 23 (57.5%) boys and 17 (42.5%) girls. The patients lived in different federal districts of the Russian Federation and neighboring countries: 13 patients from the North Caucasian FD (the Republic of Chechnya, Dagestan, Ingushetia, Karachay-Cherkessia, Kabardino-Balkaria, and Stavropol Krai), 7 from the Povolzhskiy FD (the Republic of Mordovia, Tatarstan, Bashkortostan, and Orenburg Oblast), 5 from the Central FD (Moscow, Kostroma, and Smolensk Oblast), 4 from the Siberian FD (Novosibirsk, Omsk Oblast, and the Republic of Altai), 3 from the Northwest FD (St. Petersburg and the Republic of Komi), 3 from Ukraine, 2 from the Southern FD (the Republic of Crimea and Krasnodar Krai), 1 from Belarus, and 1 with an undetermined place of residence. The average age of the examined patients was 6 years and 6 months, with a median of 6 years and 10 months (from 11 months to 16 years) at the time of the examination.

A total of 30 patients were selected for the examined group based on the selective screening carried out in the molecular genetics and medical genomics laboratory of the Federal State Budget Healthcare Institution, Central Children Clinical Hospital, from January 2016 to April 2021. The screening was carried out for 85 Russian patients aged 5 months to 8 years, with a male-to-female ratio of 2:1. The selection criteria were as follows: physical development delay, skeleton deformations, vomiting, dehydration, polyuria, metabolic acidosis, Fanconi syndrome, and photophobia. Additionally, we included ten patients with a diagnosis confirmed by using molecular genetic methods in the molecular genetics and medical genomics laboratory of the Federal State Budget Healthcare Institution, Central Children Clinical Hospital, and the DNA diagnostics laboratory of the Federal State Budgetary Institution, Research Centre for Medical Genetics.

The DNA samples of 197 healthy ethnic Karachay people from various regions of Karachay-Cherkessia and 178 ethnic Chechen people were used as a control group.

### Biochemical Testing

The cystine concentration was measured on an maXis Impact tandem mass spectrometer (Bruker, Germany). Whole blood was used as the biological material; leukocytes were extracted using the gradient method using Ficoll-Paque (Amresco, United States). Chromatographic separation was carried out on an Agilent 1260 chromatography machine (United States) using a SIELC Primesep 200 column (United States). Mass spectrometry detection was carried out in the positive ion registration mode using electrospray ionization (a thorough description of the methods is provided in Supplementary Material S1).

To determine the reference values, the cystine concentration in blood leukocytes was measured in a control group of 100 healthy donors. The male group consisted of 50 people (50%) aged 3 months to 15 years, with an average age of 8.5 years. The female group consisted of 50 people (50%) aged 2 months to 14 years, with an average age of 7 years. As an internal control, the cystine concentration was measured in seven patients (3 girls and four boys) with mutations in the *CTNS* gene. At the time of biochemical diagnostics, the patients were diagnosed based on distinct clinical features and molecular genetic analysis. The patients did not receive substrate reduction therapy.

In the healthy donor group, the cystine concentration levels varied from 0.11 to 0.45 nmol of ½cystine per milligram of protein, with a median of 0.30 nmol of ½cystine per milligram of protein, while in the group of patients with cystinosis, the cystine levels were 2.60–6.90 nmol of ½cystine per milligram of protein, with a median of 4.31 nmol of ½cystine per milligram of protein. The cutoff point was 1.0 nmol of ½cystine per milligram of protein based on the cutoff point values in international studies (Gertsman et al., 2016), the obtained reference cystine levels, and the levels in patients with cystinosis.

Two of these seven children had cystine levels of 0.15 nmol of ½cystine per milligram of protein and 0.28 nmol of ½cystine per milligram of protein, while in their genomes, we detected nucleotide variants described as pathogenic in the HGMD database, which allowed us to count these levels as false negative. An experiment showed that the cause of false-positive values was the decrease in the cystine concentration in the analyzed fraction, which could be caused by the molecule breakdown without a stabilizer. The measurement of cystine concentration in leukocytes was carried out during cell lysis via sonication with different incubation time intervals at room temperature, which showed that in 20 min of incubation, the concentration decreases by more than 50%, and in 60 min, the concentration is close to zero.

The diagnostic method was optimized as follows:

The cystine concentration was measured in polymorphonuclear leukocytes, which were obtained using a double-gradient method using HISTOPAQUE-1077 and HISTOPAQUE-1119 manufactured by SIGMA (Germany). To avoid cystine breakdown, the cellular sediment was stabilized with a solution of N-ethylmaleimide manufactured by SIGMA (Germany) following the manufacturer’s protocol described previously (Giustarini et al., 2006). The cystine concentration was measured again using the modified method in all seven internal control samples in three repetitions, with the lysis time of the analyzed fraction increased from 20 to 60 min at room temperature. All the seven samples, including the two previously noted false-positive samples, showed cystine levels above the cutoff point, even with 60 min of incubation.

### DNA Isolation and Genetic Testing

Genomic DNA was extracted using a DNA Blood Mini Kit (QIAGEN, Germany) on a QIAcube automated station (QIAGEN, Germany) following the manufacturer’s protocol. Whole blood was used as the biological material. DNA was eluted in 100 μL of DNAase-free water. The DNA quality and quantity were evaluated spectrophotometrically on a NanoPhotometer N60 spectrophotometer (Implen, Germany) and using a Qubit dsDNA HS Assay Kit for a Qubit 3.0 Fluorometer (Invitrogen, United States). The oligonucleotides for PCR were synthesized by JSC Evrogen (Russia).

Sanger sequencing was carried out using a BigDye^®^ Terminator v3.1 Cycle Sequencing Kit (Thermo Fisher Scientific, United States) following the manufacturer’s protocol. The amplification was performed on Bio-Rad T100 (Bio-Rad, United States) and ProFlex (Thermo Fisher Scientific, United States) thermocyclers. Capillary electrophoresis was carried out on ABI 3500XL and ABI 3500 (Thermo Fisher Scientific, United States) genetic analyzers. The obtained sequences were compared to the RefSeqGene references from the National Center for Biotechnology Information (NCBI) database.

To detect the 57 kb deletion, we used the multiplex PCR method suggested by Forestier et al. (1999).

To detect other gross deletions and duplications in the *CTNS* gene, we used multiplex ligase-dependent probe amplification (MLPA). The analysis was carried out using the P473 kits by MRC Holland (Netherlands).

The detection of c.1015G > A (p.G339R) and c.518A > G (p.Y173C) mutations in the *CTNS* gene was carried out during the haplotype analysis using a custom system based on allele-specific ligase reaction (probe sequences are provided in Supplementary Material S2). The results were registered via electrophoresis in polyacrylamide gel with subsequent ethidium bromide staining and UV visualization.

### Population Genetic Analysis

We examined the following four microsatellite markers from 17p13.2 (2.44-kb region around the *CTNS* gene): D17S831, D17S1798, D17S1828, D17S1876, and one intragenic marker D17S829. All the markers were chosen using the Marshfield NCBI genetic map (primer sequences are provided in Supplementary Material S3). The microsatellite markers were examined by AFLP analysis. The DNA fragments were amplified using PCR. The results were registered via electrophoresis in polyacrylamide gel with subsequent ethidium bromide staining and UV visualization.

Massive parallel sequencing (NGS) of 298 genes for four patients with a c.518A > G mutation in a homozygous state and nine non-related control samples of the Russian Federation residents of Chechen origin was used for phylogenetic analysis. The libraries for NGS were prepared using a KAPA HyperPlus Kit (Roche, United States) following the manufacturer’s protocol. The DNA fragmentation time to achieve the average fragment length of 350 bp was 15 min. Target enrichment was carried out using KAPA HyperCap hybridization probes (Roche, United States). Massive parallel sequencing was performed on the MiSeq platform (Illumina, United States) with V2 chemistry (500 cycles, paired-end reads). On average, in every run, 31.5 million reads were obtained, 88% with a Phred score higher than Q30. Bioinformatics analysis was carried out in accordance with GATK Best Practices recommendations (https://gatk.broadinstitute.org/). Genetic variants of all samples were loaded into the VCF2PopTree program (Subramanian et al., 2019) for pairwise genetic distance calculation. Based on these data, we built a phylogenetic tree.

The statistical analysis of the allele frequencies on mutant chromosomes and control group chromosomes was based on the χ2 test for a 2*2 contingency table comparing two groups: associated allele and all the other alleles. To evaluate linkage disequilibrium (LD), we used the following formula: *δ*=(PD–PN)/(1–PN), where PD is the frequency of the associated allele among mutant chromosomes and PN is the frequency of the same allele among the normal chromosomes (Bengtsson and Thomson, 1981). The confidence interval (CI) for δ was calculated as described by Diaz et al. (2000).

### Statistical Analysis

To find the association between cystine levels and *CTNS* mutation types, we used nonparametric statistical analysis methods (Mann–Whitney *U* test). The confidence calculation of differences in cystine concentration values prior to substrate reduction therapy and 6 months after the initial cystagon administration was carried out using the Wilcoxon criterion. A qualitative comparison was performed using an exact F-test.

## Results

### Clinical Results

Selective biochemical screening was carried out for 85 patients with a presumptive diagnosis of cystinosis. As a result, 30 (35.4%) examined children had cystine concentrations higher than the cutoff point: 2.6–8.9 nmol of ½cystine per milligram of protein, which is very close to the values obtained in corresponding international studies (Wilmer et al., 2011).

**FIGURE 1:**
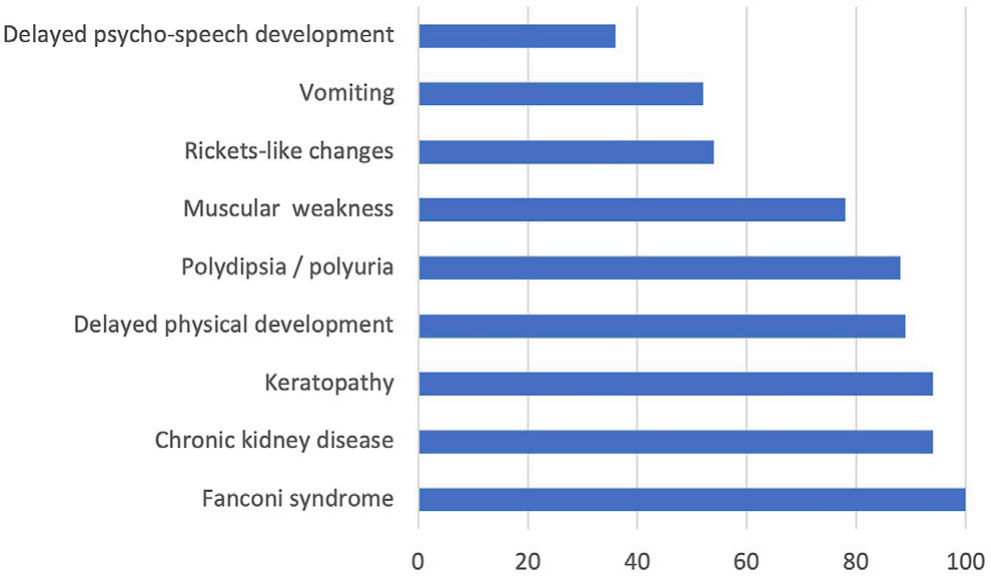
Calibration characteristics of the analytics system. The square of the calibration characteristic correlation quotient was 0.997. The lower threshold of quantitative cystine detection was 0.11 μmol/L. The cystine detection threshold was 0.01 μmol/L (Figure 2). The time of analysis for one probe was 15 min. The obtained data were processed using a built-in Bruker Data Analysis 4.1 program package.

All the examined children with available descriptions of clinical symptoms had Fanconi syndrome; 94% of them had chronic kidney disease, 94% had keratopathy, 89% had delayed physical development, 88% had polydipsia/polyuria, 78% had muscular weakness, 54% of the examined children had rickets-like changes, 52% had vomiting, and 36% had delayed psycho-speech development. The data on detected clinical features mostly corresponded to the results of international studies (Nesterova and Gahl, 2008). Only two patients (patients 11 and 16, see Supplementary Material S5) had juvenile cystinosis, while the rest had nephropathic cystinosis. The frequencies of different clinical cystinosis symptoms at the time of hospitalization after the confirmed laboratory diagnosis and before the substrate reduction therapy are presented in Figure 1.

We detected 23 different pathogenic *CTNS* variants in 40 non-related patients using various molecular genetic analysis methods. The spectra and frequencies of detected variants are presented in Table 1. The patients’ genotypes are provided in Supplementary Material S4.

**TABLE 1:** Allelic frequencies of *CTNS* (*NM_001031681.2*) GRch37 variants.

Variant	Number of chromosomes with the variant	Allelic frequency (%) (80 chromosomes)
*57 kb del*	20	25.00
c.518A>G, p.Y173C	11	13.75
c.1015G>A, p.G339R	10	12.50
c.433C>T, p.Q145*	6	7.50
c.785G>A, p.W262*	5	6.25
c.18_21del, p.Thr7Phefs*7	3	3.75
g.(?_3558266)_(3565849_?)del (ex.6-13del)	2	2.50
g.(?_3550706)_(3552123_?)del *(ex4-5del)*	2	2.50
с.699_700del, p.S234Lfs*61	2	2.50
с.451A>G, p.R151G	2	2.50
c.283G>T, p.G95*	2	2.50
g.(?_ 3558266)_( 3558736_?)del (*ex6-7del)*	2	2.50
c.140+2dup	2	2.50
c.627C>A, p.S209R	2	2.50
c.681G>A, p.E227E	1	1.25
*c.681+1G>A*	1	1.25
c.323del, p.Q108Rfs*10	1	1.25
с.613G>A, p.D205K	1	1.25
c.198_218del p.(Ile67_Pro73del)	1	1.25
c.1000del, p.T334Pfs*65	1	1.25
c.505G>T, p.G169C	1	1.25
c.413G>A, p.W138*	1	1.25
c.450G>A, p.W150*	1	1.25

It is worth noting that 13 mutations detected on 62 chromosomes (77.5%) were previously described, while 10 variants detected on 18 chromosomes (22.5%) were novel. Among the described *CTNS* variants, the 57 kb deletion was the most frequent; it was detected on 20 alleles (25.0%) in 15 (37.5%) children with infantile nephropathic cystinosis. This pathogenic variant was described in 50–60% of patients with cystinosis among European and North American residents (Anikster et al., 1999; Heil et al., 2001; Kleta et al., 2001; Kiehntopf et al., 2002). The differences in allelic frequencies of this major variant in European and Russian patients can be explained by population diversity.

The missense c.518A > G mutation leading to the p.Y173C amino acid residue replacement was previously described once (Topaloglu et al., 2012). It was detected on 11 (13.8%) alleles in children from six non-related families, five out of which are ethnically Chechen and live in the Republic of Chechnya and Ingushetia. The pathogenic c.1015G > A variant, which leads to the p.G339R amino acid residue replacement and was previously described in American, Turkish, and Iranian patients with infantile cystinosis (Topaloglu et al., 2012; Zykovich et al., 2015; Ghazi et al., 2017), was detected on 10 alleles (12.5%) in children from six non-related families of Karachay ethnicity from the Republic of Karachay-Cherkessia, Kabardino-Balkaria, and Stavropol Krai. The nucleotide variant c.433C > T, which leads to p.Q145* premature translation termination and was described in an Iranian patient (Najafi et al., 2019), was detected on five alleles in four (10.0%) patients from various regions of Russia. A previously non-described с.785G > A nucleotide variant, which leads to a p.W262* premature translation termination, was detected in four patients from three families from Tatarstan, Bashkortostan, and Moscow Oblast.

The ex.6-13del gross deletion was detected on two alleles in two non-related patients from Omsk and Smolensk Oblast. A previously non-described pathogenic c.450G > A, p.W150* variant, was detected in a homozygous state in a boy from Khabarovsk Krai. The c.413G > A, p.W138* variant was detected in a compound heterozygous state with the c.433C > T variant in a boy from the Republic of Mordovia. Both suffered from infantile nephropathic cystinosis with manifestation at the age of 7 months.

A boy with severe infantile nephropathic cystinosis had a previously non-described single-nucleotide deletion c.1000del leading to a p.T334Pfs*65 frameshift in a compound heterozygous state with 57 kb deletion. A missense c.627C > A, p.S209R variant was detected in a homozygous state in a boy with juvenile nephropathic cystinosis from the Republic of Bashkortostan. This variant has not been described in the gnomAD database (version 2.1.1) and was not present in exomes of 1337 Russian patients with various referral diagnoses. Overall, 10 programs (BayesDel_addAF, DANN, DEOGEN2, FATHMM-MKL, LIST-S2, M-CAP, MVP, MutationAssessor, MutationTaster, and SIFT) predict the pathogenicity of this variant. The patient had distinct clinical and biochemical data in favor of the “nephropathic cystinosis” diagnosis; thus, the variant was interpreted as likely pathogenic. Another previously non-described variant—a gross deletion including exons 6 and 7 of the *CTNS* gene—was detected in a homozygous state in a patient from Ukraine.

A female patient from Belarus also suffering from juvenile nephropathic cystinosis had a previously non-described с.140+2dup duplication in a homozygous state. This variant was present on one chromosome out of 251,172 in the gnomAD database (version 2.1.1) and not present in the 1337 Russian exomes. The variant, according to prediction programs (NETGENE2 and HSF), is very likely to affect the donor splice site of exon 4 (NM_001031681.2). However, despite the typical clinical and biochemical phenotypes of the patient, this variant was interpreted as VUS.

Two Ukrainian patients had three previously described (Shotelersuk et al., 1998; Town et al., 1998; Mason et al., 2003) pathogenic variants: с.699_700del, p.S234Lfs*61 in a homozygous state and c.18_21del, p.T7Ffs*7 in compound heterozygous state with с.613G > A, p.D205K.

**FIGURE 2:**
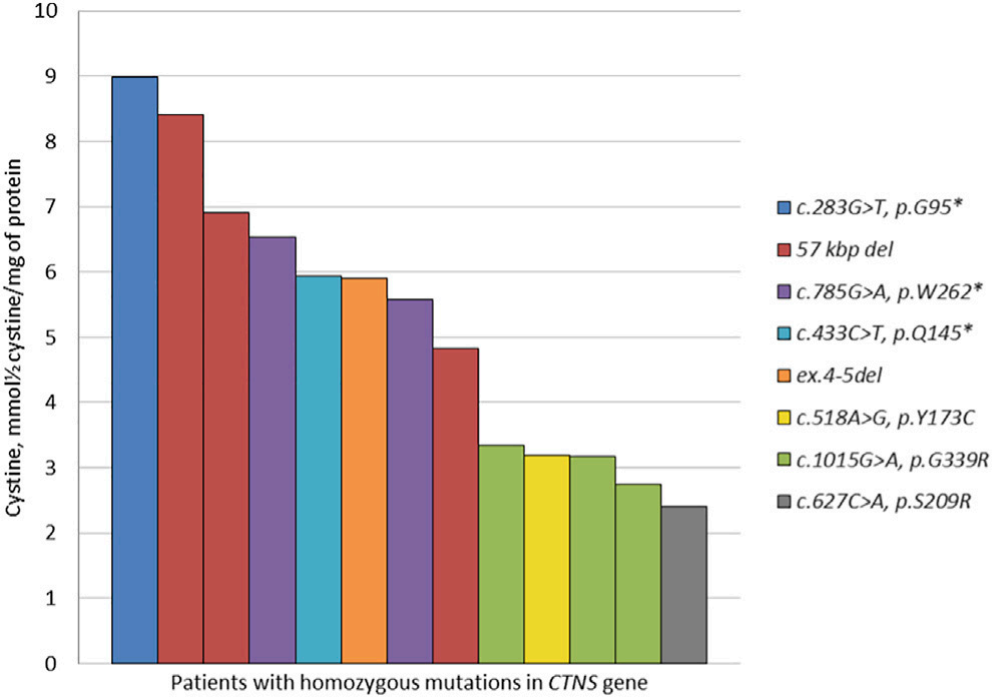
Chromatogram of the standard cystine solution with a concentration of 0.11 μmol/L corresponding to the lower threshold of the quantitative detection method.

A high percentage of homozygous *CTNS* variants allowed us to describe the correlations of certain mutation types with blood cystine levels prior to the substrate reduction therapy. The lowest concentration was detected in a patient with a missense variant c.627C > A, p.S209R in a homozygous state, while the patient with a nonsense c.283G > T, p.G95* variant in a homozygous state showed the highest cystine concentration (Figure 2).

Based on the analysis of the obtained data, we detected statistically valid differences (*p* = 0.003) between high cystine concentrations in a group of patients with homozygous LoF variants (the 57 kb deletion, nonsense c.283G > T, c.433C > T, c.785G > A mutations) compared to the group of patients with homozygous missense c.518A > G, c.627C > A, c.1015G > A mutations in the *CTNS* gene (Table 2).

**TABLE 2:** Cystine levels in groups of patients with different *CTNS* mutation types.

Parameter	Patients with homozygous missense mutations	Patients with homozygous Lof mutations	p-value (Mann–Whitney U test)
Cystine concentration, ½cystine per milligram of protein	3.2 (2.9–3.2)	6.3 (5.9–7.3)	0.003

*Note:* Cystine concentrations are presented as median values and quartiles (25%–75%).

The patients responded adequately to pathogenetic therapy with cysteamine bitartrate in most cases, which is confirmed by normal—0.78 (0.2–1.1)—levels of cystine measured 6 months after the start of the therapy. The excess cystine level detected in two cases was caused by a break from taking the medication in one child and the necessity of dose correction in another. Considering those factors, the treatment was corrected, and both patients showed normal cystine levels at the time of the next hospitalization.

Considering that nephropathic cystinosis has autosomal recessive inheritance, this condition might prevail in some ethnic and geographic isolates because of local founder effects or the high number of inbred marriages. In the examined patient groups, we detected a high frequency of the c.1015G > A pathogenic variant in Karachay residents and the c.518A > G variant in Chechen residents.

Kinship of probands with the same mutations was excluded via questioning relatives in at least three generations.

The c.1015G > A variant was the main cause of nephrotic cystinosis in ethnic Karachay patients: four out of six patients with this variant were Karachay-Cherkessia residents and the other two were from neighboring regions: Stavropol Krai and the Republic of Kabardino-Balkaria. All families of patients with this variant were ethnically Karachay.

### Population Genetic Analysis Results

We analyzed the five following microsatellite markers from the *CTNS* gene region: D17S831, D17S1798, D17S1828, D17S1876, and D17S829; haplotype analysis was carried out on the material of four non-related patients homozygous on the c.1015G > A variant and two compound heterozygous: c[1015G > A]; [18_21del] and c[1015G > A]; [681G > A]. The genotyping results are presented in Table 3. The presumed founder haplotype is highlighted with gray.

**TABLE 3:** Haplotypes of chromosomes with the c.1015G>A mutation for markers D17S831-D17S1798-D17S829-D17S1828-D17S1876.

Patient	Marker	**D17S831**	**D17S1798**	**CTNS**	**D17S829**	**D17S1828**	**D17S1876**
	Coordinate (kB)	1.910	2.706	3.540-3.566	3.550	3.810	4.345
	Place of residence						
BM	Republic of Karachay-Cherkessia	1	1	c.1015G>A	3	3	9
		1	1	c.1015G>A	3	3	9
SF	Republic of Karachay-Cherkessia	1	1	c.1015G>A	3	3	9
		8	3	c.1015G>A	3	3	10
TSR	Republic of Karachay-Cherkessia	1	1	c.1015G>A	3	3	9
		1	1	c.1015G>A	3	3	9
KI	Republic of Karachay-Cherkessia	3	1	c.1015G>A	3	3	9
		3	7	c.18_21del	7	3	8
BRA	Republic of Kabardino-Balkaria	3	1	c.1015G>A	3	3	7
		7	2	c.681G>A	7	4	6
ESA	Republic of Karachay-Cherkessia	1	2	c.1015G>A	3	3	9
		1	2	c.1015G>A	3	3	9

As a control group, we typed DNA samples of 18 non-related Karachay people without the c.1015G > A mutation on the same microsatellite markers. The allelic frequencies in eight mutant (D) chromosomes of homozygous patients and 38 control chromosomes (N) for five microsatellite loci above (D17S831, D17S1798), below (D17S1828, D17S1876), and in the intron (D17S829) of the *CTNS* gene on a Marshfield genetic map are provided in Supplementary Material S6. F-test results for alleles of markers with the highest linkage disequilibrium parameter δ are presented in Table 4.

**TABLE 4:** Linkage disequilibrium analysis between *CTNS* c.1015G>A mutation and microsatellites closest to the *CTNS* gene.

Marker	Coordinate cM	Allele	p-value	δ±95 CI
D17S831	6.60	1	p<0.05	0.864±0.256
D17S1798	6.60	1	p>0.05	0.289±0.694
D17S829 (CTNS)	10.02	3	—	—
D17S1828	10.02	3	—	—
D17S1876	10.72	9	p<0.05	0.868±0.248

Thus, the 1-1-3-3-9 haplotype on markers D17S831-D17S1798-D17S829-D17S1828-D17S1876 is highly likely to be the founder haplotype subjected to gradual decay, and the accumulation of the c.1015G > A mutation in the *CTNS* gene in the Republic of Karachay-Cherkessia is caused by the founder effect.

We scanned DNA samples of 197 healthy Karachay residents from various regions of the Republic of Karachay-Cherkessia for the c.1015G > A mutation and did not detect any carriers of this variant. Thus, despite the undoubted presence of the founder effect for this mutation, its carrier frequency is not very high on the territory of the Republic of Karachay-Cherkessia, and the accumulation of patients with this mutation is most likely caused by inbred marriages, although the parents of the probands with the *CTNS* variant in a homozygous state deny kinship.

The c.518A > G variant was the most common among patients from the Republic of Chechnya: four patients from this republic had the mutation in a homozygous state, and an additional patient from Ingushetia, which borders Chechnya. Outside of the Republic of North Caucasus, the c.518A > G mutation was detected in a compound heterozygous state with the common 57 kb deletion in one Russian patient from the Moscow Oblast. Four homozygous samples were genotyped using the following microsatellite markers: D17S831-D17S1798-D17S829-D17S1828-D17S1876 (Table 5).

**TABLE 5:** Haplotypes of chromosomes with the c.518A > G mutation for markers D17S831-D17S1798-D17S829-D17S1828-D17S1876.

Patient	Marker	D17S831	D17S1798	CTNS	D17S829	D17S1828	D17S1876
—	Coordinate (kB)	1.910	2.706	3.540–3.566	3.550	3.810	4.345
	Place of residence						
MM	Chechnya	4	1	c.518A > G	1	5	7
		4	1	c.518A > G	1	5	7
MSU	Chechnya	6	1	c.518A > G	1	5	7
		6	2	c.518A > G	1	2	1
IAM	Chechnya	9	2	c.518A > G	1	8	7
		1	1	c.518A > G	1	5	7
EIS	Ingushetia	3	2	c.518A > G	1	8	7
		3	3	c.518A > G	1	5	7

The control group consisted of 17 non-related Chechnya residents without the c.518A > G mutation. The genotyping results are presented in Table 5. The presumed founder haplotype is highlighted with gray. The frequencies of alleles carrying the mutation on homozygous patients’ chromosomes and control chromosomes for five microsatellite loci above (D17S831, D17S1798), below (D17S1828, D17S1876), and in the intron (D17S829) of the *CTNS* gene on the Marshfield genetic map are provided in Supplementary Material S7.

The F-test results for alleles of markers with the highest linkage disequilibrium values (*δ*) are presented in Table 6.

**TABLE 6:** Linkage disequilibrium analysis between *CTNS* c.518A > G mutation and microsatellites closest to the *CTNS* mgene.

Marker	**Coordinate cM**	Allele	*p*-value	δ±95 CI
D17S831	6.60	6	*p* > 0.05	0.177 ± 0.348
D17S1798	6.60	—	—	—
D17S829 (CTNS)	10.02	1	—	—
D17S1828	10.02	5	*p* > 0.05	0.469 ± 0.502
D17S1876	10.72	9	*p* < 0.05	0.863 ± 0.257

Even though the founder effect is traceable for this mutation, the decay of this genotype is very strong, starting in the genetic coordinates of the *CTNS* gene at marker D17S1828. It is possible that later two more local founder effects caused the two genotype groups on markers D17S1798-D17S829-D17S1828-D17S1876: 1-1-5-7 and 2-1-8-7.

During the analysis of 178 DNA samples of ethnic Chechen people, we detected one carrier of the c.518A > G mutation in a heterozygous state.

**FIGURE 3:**
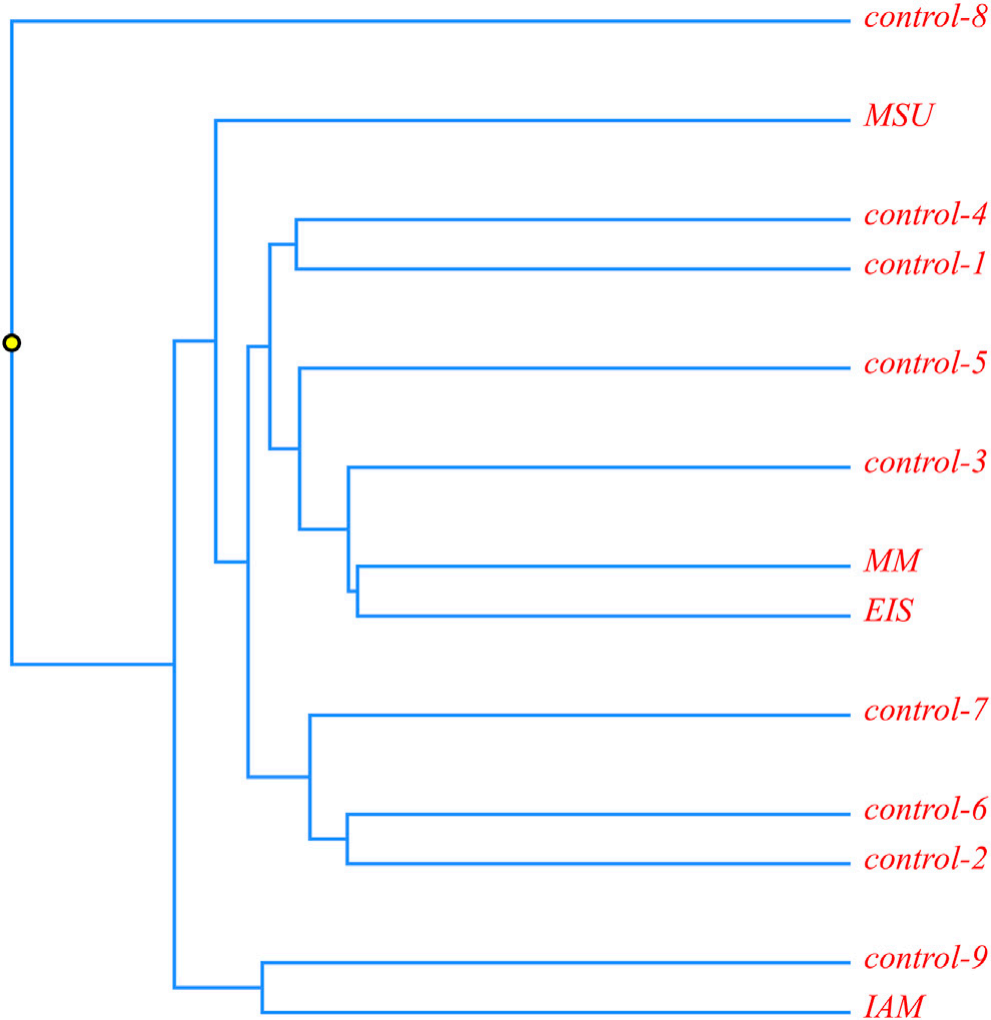
Phylogenetic analysis of ethnic Chechen children with the c.518A>G pathogenic variant; and control samples without it.

A phylogenetic analysis was carried out for four patients with the c.518A > G mutation in a homozygous state. It was suggested that in case of a founder effect, the genetic distance between the samples would be minimal, and on phylogenetic trees, these samples will form a separate cluster. The tree diagram illustrating the genetic distance between the samples is presented in Figure 3.

It is obvious that the samples with the homozygous c.518A > G mutation are located in different clusters and are not isolated from the control samples. This allows us to suggest that this mutation could appear in several isolates during an insignificant time frame or that its accumulation is a result of inbred marriages.

## Discussion

More than 90% of detected cystinosis cases are caused by a 57 kb deletion and mutations located in exons 7–10 and 12a of the *CTNS* gene, which mostly corresponds with international studies (Anikster et al., 1999; Forestier et al., 1999). Among the previously described *CTNS* variants, the most frequent was the 57257-bp deletion, which was found on 20 alleles (25.0%) in 15 (37.5%) children with infantile cystinosis. The differences in frequencies of this major variant among European and Russian patients can be explained by the prevalence of patients from the Republic of Chechnya (4/11.4%) and the Republic of Karachay-Cherkessia (4/11.4%) in the examined group. In the North Caucasian Republic, the c.518A > G mutation in the Chechen ethnos and the c.1015G > A in the Karachay ethnos are accumulated as a result of local founder effects and inbred marriages.

In this study, we detected ten novel variants of the *CTNS* gene, eight of which are classified as pathogenic, one as likely pathogenic, and one as VUS in accordance with the Russian guide for the interpretation of the human DNA sequence data (Рыжкова et al., 2019). A previously non-described с.785G > A (p.W262*) nucleotide variant was detected in patients from three families; it also shows the tendency to accumulate in patients from Povolzhye. A previously non-described ex.6-13del gross deletion was detected in two non-related patients. Each of the remaining novel mutation was detected once.

In total, among 23 detected variants in the *CTNS* gene, LoF variants prevailed: nine (39.1%) deletions, including four gross deletions (18%), five (21.7%) nonsense mutations, and one (4.4%) canonic splice site mutation. Aside from that, we detected seven (30.4) pathogenic and likely pathogenic missense mutations and one VUS variant affecting the canonic splice site. According to the information presented in the HGMD professional database on the types of detected mutations in the *CTNS* gene, missense and nonsense mutations had the highest percentage (41%). However, there were differences as well. Thus, a significant percentage (18%) of gross deletions detected in this study is slightly higher than that in the HGMD database, where this value is 12%. On the other side, the percent of canonic splice site mutations described in the literature is slightly higher (14%). These differences are likely to be caused by the small number of patients in our cohort compared to the worldwide data in the HGMD professional database.

The patients presented in this study mostly had infantile nephropathic cystinosis and responded to cysteamine bitartrate pathogenetic therapy, which is confirmed by multiple measurements of cystine levels during the therapy monitoring. We showed the difference between the mutation type (LoF or missense) and blood cystine levels prior to the pathogenetic therapy. Studies of international scientists describing various population groups demonstrate the association of mutations that prematurely interrupt the protein synthesis with early manifestation and severe clinical picture of cystinosis (Taranta et al., 2010; Al-Haggar, 2013; Alcántara-Ortigoza et al., 2013; Ferreira et al., 2018). According to these data, we can assume that high cystine levels may correlate directly with the severity of cystinosis and may be an unfavorable prognostic factor for patients with LoF mutations in the *CTNS* gene.

## Conclusion

The genetic landscape of nephropathic cystinosis in Russian patients shows the major variants c.1015G > A and c.518A > G to be characteristic for Karachay and Chechen ethnic groups.

Nephropathic cystinosis is an orphan disease of metabolic nature, which requires a complex approach for its diagnosis. The practice of screening the population for cystinosis by biochemical testing is currently technically complicated due to high blood volume and short lifetime of polymorphonuclear leukocytes; therefore, the effort aimed at the reduction of the number of disease cases should be primarily focused on selecting patients into the risk group at the youngest age, as well as on cascade analysis of relatives in affected families.

An early diagnostics using the methods presented in this study and the early start of complex therapy combining symptomatic and pathogenetic substrate reduction therapy, continuing the latter during the patient’s life, have significant importance in establishing the best control over the disease, preventing the growth deceleration, and delaying chronic kidney disease and other renal and extrarenal complications.

## Data Availability

The datasets presented in this study can be found in online repositories. The names of the repository/repositories and accession number(s) can be found at: https://www.ncbi.nlm.nih.gov/, PRJNA804010.
