# Supplementary material for: Genetic Landscape of Nephropathic Cystinosis in Russian Children
Source: Front Genet. 2022 Apr 28;13:863157. doi: 10.3389/fgene.2022.863157 (PMC9096100; doi:10.3389/fgene.2022.863157)
Supplement: Supplementary file 1 [file DataSheet1.PDF]

Supplementary 1

Cystine concentration measurement

The cystine concentration measurement was carried out on a Bruker Maxis Impact tandem mass spectrometer (Germany). Whole blood was used as biological material; leukocytes were obtained via the gradient method using Ficoll-Paque (Amresco, USA). Chromatographic separation was carried out on an Agilent 1260 chromatography machine (USA) using a SIELC Primesep 200 column (USA). DNA was eluted using a mixture of acetonitrile and water with added formic acid. The reagents had grades appropriate for high-performance liquid chromatography (HPLC). Mass spectrometric detection was carried out in the anion detection mode using electrospray ionization. The mass analyzer was used in the ion detection mode (100–500 m/z) with mass detection precision minimum of 5 ppm and resolution minimum of 20000 (FWHM). The analytic system was calibrated within a range of cystine concentrations of 0.11–11.10 µmol/l (Figure 1).

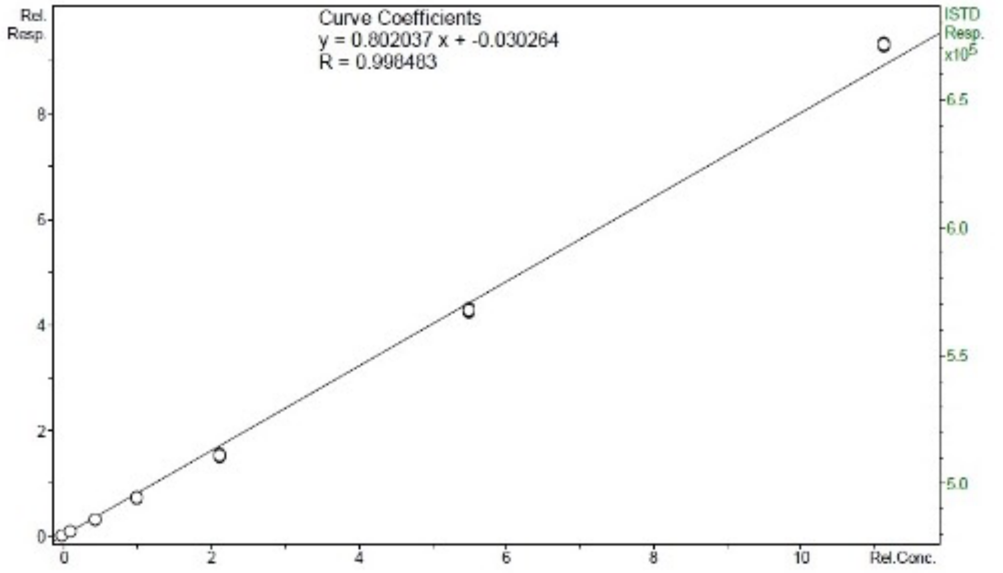

Figure 1. Calibration characteristics of the analytic system.

The square of the calibration characteristic correlation quotient was 0.997. The lower threshold of quantitative cystine detection was 0.11 µmol/l. The cystine detection threshold was 0.01 µmol/l. (Figure 2). The time of analysis for one probe was 15 minutes. The obtained data was processed using a built-in Bruker Data Analysis 4.1 program package.

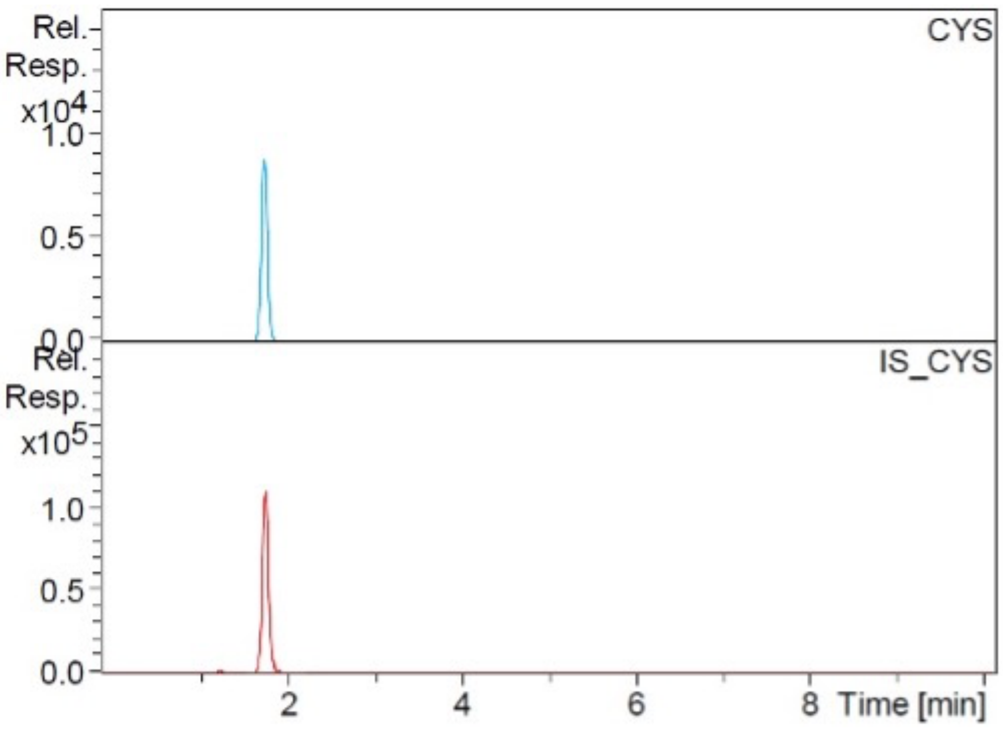

Figure 2. Chromatogram of the standard cystine solution with the concentration of 0.11 µmol/l corresponding with the lower threshold of the quantitative detection method.

Supplementary 2

Probe sequences for detection of mutations c.1015G>A (p.G339R) и c.518A>G (p.Y173C) in the *CTNS* gene.

mctns518fn-GTTCGTACGTGAATCGCGGTACTCTGACGGGCTTCGTGGCCTA,

mctns518fm-GTTCGTACGTGAATCGCGGTACGACGGGCTTCGTGGCCTG,

mctns518r-CAGTGTATTCAACATCGGCCTCC GATGCGATCCGATGCCTTCATG;

mctns1015fn-GTTCGTACGTGAATCGCGGTACGTTGACCCAACCAAGTTTGGACTCG,

mctns1015fm-GTTCGTACGTGAATCGCGGTACGACCCAACCAAGTTTGGACTCA,

mctns1015r-GGGTCTTCTCCATCGTCTTCGTTTTCGATGCGATCCGATGCCTTCATG.

Supplementary 3

The following primer sequences were used for marker amplification:

D17S831: F-CTGCAGACCTAGGCCATGTTC,

R- CGGGACTTGAATTACTATCATCC,

D17S1798: F-GACCTTGATCATATCTTGATCATC,

R- GATTTCCAGAATGAAATTGCTGATC,

D17S1828: F-CAGCCACACACCCCATACCC,

R- CCCTTCCTCGAGGAAAGACTC,

D17S1876: F-GACCTTTCTCTTCACCTACTGC,

R- GTATGGTTCTTACCGTAGTGGTC,

D17S829: F-CTAGGGGAGCGTGTTAGCATTAC,

R- CTGGAGCATGTGCGTGTGCC.

Supplementary 4

Table 1. Patients' genotypes

N	sex	Place of residence	*Variant 1*	*Description (HGMD professional)*	*Variant 2*	*Description (HGMD professional)*
1	F	Mordovia	*57 kb deletion*	CG004969	*57 kb deletion*	CG004969
2	F	Karachay-Cherkessia	*c.1015G>A, p.G339R*	CM980461	*c.1015G>A, p.G339R*	CM980461
3	F	Karachay-Cherkessia	*c.1015G>A, p.G339R*	CM980461	*c.1015G>A, p.G339R*	CM980461
4	F	Stavropol Krai	*c.1015G>A, p.G339R*	CM980461	*c.1015G>A, p.G339R*	CM980461
5	F	Chechnya	*c.518A>G, p.Y173C*	CM1110322	*c.518A>G, p.Y173C*	CM1110322
6	M	Karachay-Cherkessia	*c.18_21del, p.Thr7Phefs*7*	CD982561	*c.1015G>A, p.G339R*	CM980461
7	M	Khabarovsk Krai	*57 kb deletion*	CG004969	*c.450G>A, p.W150**	n/a
8	M	Altai Krai	*c.283G>T, p.G95**	CD982561	*c.283G>T, p.G95**	CD982561
9	M	Crimea	*57 kb deletion*	CG004969	*c.1000del, p.T334Pfs*65*	n/a
10	M	Krasnodar Krai	*57 kb deletion*	CG004969	*c.18_21del, p.T7Ffs*7*	CD982561
11	F	Belarus	*c.140+2dup*	n/a	*c.140+2dup*	n/a
12	F	Republic of Kabardino-Balkaria	*57 kb deletion*	CG004969	*57 kb deletion*	CG004969
13	M	Moscow Oblast	*c.433C>T, p.Q145**	CM195019	*c.433C>T, p.Q145**	CM195019
14	M	Orenburg Oblast	*c.433C>T, p.Q145**	CM195019	*c.681+1G>A*	CS993011
15	F	Tatarstan	*57 kb deletion*	CG004969	*c.785G>A, p.W262**	n/a
16	F	n/a	*57 kb deletion*	CG004969	*57 kb deletion*	CG004969
17	F	Tatarstan	*c.785G>A, p.W262**	n/a	*c.785G>A, p.W262**	n/a
18	M	Komi	*57 kb deletion*	CG004969	*c.433C>T, p.Q145**	CM195019
19	M	Bashkortostan	*57 kb deletion*	CG004969	*c.785G>A, p.W262**	n/a
20	M	Moscow Oblast	*57 kb deletion*	CG004969	*c.785G>A, p.W262**	n/a
21	M	Bashkortostan?	*c.627C>A, p.S209R*	n/a	*c.627C>A, p.S209R*	n/a
22	F	Chechnya	*c.518A>G, p.Y173C*	CM1110322	*c.518A>G, p.Y173C*	CM1110322
23	M	Novosibirsk Oblast	*57 kb deletion*	CG004969	*c.505G>T, p.G169C*	n/a

24	F	Chechnya	*c.518A>G, p.Y173C*	CM1110322	*c.518A>G, p.Y173C*	CM1110322
25	M	Mordovia	*c.413G>A, p.W138**	n/a	*c.433C>T, p.Q145**	CM195019
26	M	Saint-Petersburg	*57 kb deletion*	CG004969	*c.323del, p.Q108Rfs*10*	CD172137
27	M	Moscow Oblast	*57 kb deletion*	CG004969	*c.518A>G, p.Y173C*	CM1110322
28	F	Kostroma	*57 kb deletion*	CG004969	*57 kb deletion*	CG004969
29	M	Chechnya	*c.518A>G, p.Y173C*	CM1110322	*c.518A>G, p.Y173C*	CM1110322
30	M	Republic of Kabardino-Balkaria	*c.1015G>A, p.G339R*	CM980461	*c.681G>A, p.E227E*	CS099126
31	M	Novosibirsk Oblast	*57 kb deletion*	CG004969	*57 kb deletion*	CG004969
32	M	Ingushetia	*c.518A>G, p.Y173C*	CM1110322	*c.518A>G, p.Y173C*	CM1110322
33	M	Dagestan	*g.(?_3550706)_(3552123_?)del*	CG994863	*g.(?_3550706)_(3552123_?)del*	CG994863
34	F	Karachay-Cherkessia	*c.1015G>A, p.G339R*	CM980461	*c.1015G>A, p.G339R*	CM980461
35	M	Ukraine	*g.(?_ 3558266)_(3558736_?)del*	n/a	*g.(?_3558266)_(3558736_?)del*	n/a
36	F	Saint-Petersburg	*c.451A>G, p.R151G*	CM1110322	*c.451A>G, p.R151G*	CM1110322
37	F	Ukraine	*c.18_21del, p.T7Ffs*7*	CD982561	*c.613G>A, p.D205K*	CM980461
38	M	Ukraine	*c.699_700del, p.S234Lfs*61*	CD031495	*c.699_700del, p.S234Lfs*61*	CD031495
39	M	Omsk Oblast	*c.198_218del p.(Ile67_Pro73del)*	n/a	*g.(?_3558266)_(3565849_?)del*	n/a
40	F	Smolensk Oblast	*c.433C>T, p.Q145**	CM195019	*g.(?_3558266)_(3565849_?)del*	n/a

Supplementary 5

Table 2. Clinical features of patients

N	Sex	Age of onset, months	Vomiting	Hepatomegaly	Splenomegaly	Muscle weakness	Polydipsia / Polyuria	Failure to thrive	Developmental delay	Chronic kidney disease at the time of diagnosis	Fanconi syndrome (secondary)	Eye injury (keratitis)	Rickets-like changes
1	F	6	yes	no	no	yes	yes	yes	yes	1	yes	yes	no
2	F	3,5	yes	yes	no	yes	yes	yes	yes	2	yes	yes	no
3	F	3	no	yes	yes	yes	yes	yes	no	1	yes	yes	no
4	F	6	yes	yes	no	yes	yes	yes	no	2	yes	yes	no
5	F	4	yes	no	no	yes	yes	yes	yes	1	yes	yes	yes
6	M	5	no	yes	no	yes	yes	yes	no	1	yes	yes	no
7	M	7	no	yes	no	yes	yes	yes	yes	1	yes	yes	yes
8	M	12	yes	no	no	yes	yes	yes	yes	1	yes	yes	yes
9	M	18	yes	no	no	no	yes	no	no	5	yes	yes	yes
10	M	12	yes	n/a	n/a	yes	yes	yes	yes	5	yes	yes	no
11	F	144	no	n/a	n/a	yes	no	yes	no	no	yes	yes	no
12	F	6	n/a	yes	yes	no	yes	yes	no	1	yes	yes	yes
13	M	4	yes	n/a	n/a	yes	yes	yes	yes	no	yes	yes	no
14	M	18	no	yes	no	yes	yes	yes	no	1	yes	yes	yes
15	F	15	yes	no	no	no	yes	yes	no	2	yes	yes	yes
16	F	132	n/a	n/a	n/a	no	yes	yes	no	no	yes	yes	no

17	F	6	no	no	no	yes	yes	yes	yes	3	yes	yes	no
18	M	3	no	yes	no	yes	yes	yes	no	1	yes	yes	no
19	M	6	yes	yes	no	yes	yes	yes	yes	3	yes	yes	yes
20	M	10	no	yes	no	no	no	no	no	no	yes	no	no
21	M	18	no	no	no	yes	yes	yes	no	5	yes	yes	yes
22	F	16	yes	yes	no	yes	yes	yes	yes	1	yes	yes	n/a
23	M	12	no	no	no	no	n/a	yes	no	5	yes	yes	yes
24	F	18	yes	yes	no	yes	yes	yes	yes	4	yes	yes	yes
25	M	7	no	no	no	yes	yes	yes	no	no	yes	yes	yes
26	M	2	yes	yes	yes	yes	yes	yes	yes	5	yes	yes	yes
27	M	4	нет	no	no	yes	no	yes	no	1	yes	yes	no
28	F	7	н/д	n/a	n/a	yes	yes	yes	yes	n/a	yes	yes	no
29	M	15	no	yes	yes	yes	yes	yes	no	1	yes	no	yes
30	M	8	yes	yes	yes	yes	yes	no	no	1	yes	yes	yes
31	M	6	yes	yes	yes	yes	yes	yes	no	2	yes	yes	yes
32	M	7	yes	yes	yes	yes	yes	yes	no	1	yes	yes	yes
33	M	7	no	no	no	yes	yes	yes	n/a	3	yes	yes	yes
34	F	12	yes	no	no	yes	yes	yes	no	4	yes	yes	yes
35	M	n/a	n/a	n/a	n/a	n/a	n/a	n/a	n/a	n/a	n/a	n/a	n/a
36	F	n/a	n/a	n/a	n/a	n/a	n/a	n/a	n/a	n/a	n/a	n/a	n/a
37	F	n/a	n/a	n/a	n/a	n/a	n/a	n/a	n/a	n/a	n/a	n/a	n/a
38	M	n/a	n/a	n/a	n/a	n/a	n/a	n/a	n/a	n/a	n/a	n/a	n/a

39	M	72	no	no	no	no	no	no	no	5	yes	yes	no
40	F	18	no	no	no	no	yes	yes	no	4	yes	yes	no

Supplementary 6

Table 3. Allele frequencies of microsatellite markers on chromosomes with the c.1015G>A mutation (D) and on chromosomes without the mutation (N).

Marker	D17S831		D17S1798		D17S829 (CTNS)		D17S1828		D17S1876	
	D	N	D	N	D	N	D	N	D	N
Allele	Number of alleles/ frequencies (%)									
1	**7/87.5**	3/8.3	**5/62.5**	17/47.2		10/27.8		1/2.7		5/13.9
2			2/25	16/44.4		1/2.7		9/25.0		8/22.2
3		9/25.0	1/12.5	1/2.7	**8/100**	12/33.3	**8/100**	12/33.3		4/11.1
4		13/36.1		1/2.7		13/36.1		3/8.3		
5		1/2.7		1/2.7				8/22.2		2/5.6
6		3/8.3						1/2.7		6/16.7
7		3/8.3								6/16.7
8	1/12.5	2/5.6						1/2.7		3/8.3
9		1/2.7						1/2.7	**7/87.5**	2/5.6
10		1/2.7							1/12.5	
Number of examined chromosomes	8	36	8	36	8	36	8	36	8	36

The frequencies of alleles that showed maximal linkage disequilibrium with the c.1015G>A mutation are underlined.

Supplementary 7

Table 4. Allele frequencies of microsatellite markers on chromosomes with the c.518A>G mutation (D) and on chromosomes without the mutation (N).

Marker	D17S831		D17S1798		D17S829 (CTNS)		D17S1828		D17S1876	
	D	N	D	N	D	N	D	N	D	N
Allele	Number of alleles/ frequencies (%)									
1	1/12.5	5/14.7	4/50.0	16/50.0	**8/100**	10/29.4		1/2.9	1/12.5	9/26.5
2		1/2.9	3/37.5	13/40.6		1/2.9	1/12.5	3/8.8		1/2.9
3	2/25.0	9/26.5	1/12.5			1/2.9		5/14.7		
4	2/25.0	5/14.7				19/55.9		4/11.8		
5		1/2.9		1/3.1			**5/62.5**	10/29.4		2/5.9
6	**2/25.0**	3/8.8				1/2.9		2/5.9		4/11.8
7		5/14.7						1/2.9	**7/87.5**	3/8.8
8		3/8.8				1/2.9	2/25.0	2/5.9		6/17.6
9	1/12.5	1/2.9		2/6.3				4/11.8		4/11.8
10						1/2.9				5/14.7
11		1/2.9						2/5.9		
Number of examined chromosomes	8	34	8	32	8	34	8	34	8	34

The frequencies of alleles that showed maximal linkage disequilibrium with the c.518A>G mutation are underlined.
