# Supplementary material for: Genetic Landscape of Nephropathic Cystinosis in Russian Children
Source: Front Genet. 2022 Apr 28;13:863157. doi: 10.3389/fgene.2022.863157 (PMC9096100; doi:10.3389/fgene.2022.863157)
Supplement: Supplementary file 2 [file DataSheet2.DOCX]

**Supplementary 1**

Cystine concentration measurement

The cystine concentration measurement was carried out on a Bruker Maxis Impact tandem mass spectrometer (Germany). Whole blood was used as biological material; leukocytes were obtained via the gradient method using Ficoll-Paque (Amresco, USA). Chromatographic separation was carried out on an Agilent 1260 chromatography machine (USA) using a SIELC Primesep 200 column (USA). DNA was eluted using a mixture of acetonitrile and water with added formic acid. The reagents had grades appropriate for high-performance liquid chromatography (HPLC). Mass spectrometric detection was carried out in the anion detection mode using electrospray ionization. The mass analyzer was used in the ion detection mode (100–500 m/z) with mass detection precision minimum of 5 ppm and resolution minimum of 20000 (FWHM). The analytic system was calibrated within a range of cystine concentrations of 0.11–11.10 µmol/l (Figure 1).


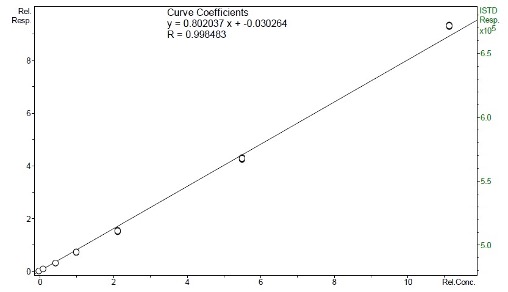


**Figure 1.** Calibration characteristics of the analytic system.

The square of the calibration characteristic correlation quotient was 0.997. The lower threshold of quantitative cystine detection was 0.11 µmol/l. The cystine detection threshold was 0.01 µmol/l. (Figure 2). The time of analysis for one probe was 15 minutes. The obtained data was processed using a built-in Bruker Data Analysis 4.1 program package.


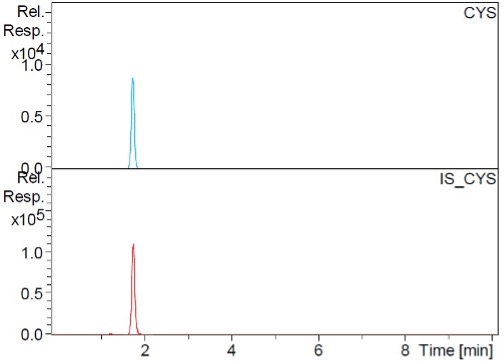


**Figure 2.** Chromatogram of the standard cystine solution with the concentration of 0.11 µmol/l corresponding with the lower threshold of the quantitative detection method.

**Supplementary 2**

Probe sequences for detection of mutations c.1015G>A (p.G339R) и c.518A>G (p.Y173C) in the *CTNS* gene.

mctns518fn-GTTCGTACGTGAATCGCGGTACTCTGACGGGCTTCGTGGCCTA, mctns518fm-GTTCGTACGTGAATCGCGGTACGACGGGCTTCGTGGCCTG,

mctns518r-CAGTGTATTCAACATCGGCCTCC GATGCGATCCGATGCCTTCATG;

mctns1015fn-GTTCGTACGTGAATCGCGGTACGTTGACCCAACCAAGTTTGGACTCG, mctns1015fm-GTTCGTACGTGAATCGCGGTACGACCCAACCAAGTTTGGACTCA,

mctns1015r-GGGTCTTCTCCATCGTCTTCGTTTTCGATGCGATCCGATGCCTTCATG.

**Supplementary 3**

The following primer sequences were used for marker amplification:

D17S831: F-CTGCAGACCTAGGCCATGTTC,

R- CGGGACTTGAATTACTATCATCC,

D17S1798: F-GACCTTGATCATATCTTGATCATC,

R- GATTTCCAGAATGAAATTGCTGATC,

D17S1828: F-CAGCCACACACCCCATACCC,

R- CCCTTCCTCGAGGAAAGACTC,

D17S1876: F-GACCTTTCTCTTCACCTACTGC,

R- GTATGGTTCTTACCGTAGTGGTC,

D17S829: F-CTAGGGGAGCGTGTTAGCATTAC,

R- CTGGAGCATGTGCGTGTGCC.

**Supplementary 4**

**Table 1.** Patients' genotypes

| **N** | **sex** | **Place of residence** | ***Variant 1*** | ***Description***  ***(HGMD professional)*** | ***Variant 2*** | ***Description***  ***(HGMD professional)*** |
| --- | --- | --- | --- | --- | --- | --- |
| 1 | F | Mordovia | *57 kb deletion* | CG004969 | *57 kb deletion* | CG004969 |
| 2 | F | Karachay-Cherkessia | *c.1015G>A, p.G339R* | CM980461 | *c.1015G>A, p.G339R* | CM980461 |
| 3 | F | Karachay-Cherkessia | *c.1015G>A, p.G339R* | CM980461 | *c.1015G>A, p.G339R* | CM980461 |
| 4 | F | Stavropol Krai | *c.1015G>A, p.G339R* | CM980461 | *c.1015G>A, p.G339R* | CM980461 |
| 5 | F | Chechnya | *c.518A>G, p.Y173C* | CM1110322 | *c.518A>G, p.Y173C* | CM1110322 |
| 6 | M | Karachay-Cherkessia | *c.18_21del, p.Thr7Phefs*7* | CD982561 | *c.1015G>A, p.G339R* | CM980461 |
| 7 | M | Khabarovsk Krai | *57 kb deletion* | CG004969 | *c.450G>A, p.W150** | n/a |
| 8 | M | Altai Krai | *c.283G>T, p.G95** | CD982561 | *c.283G>T, p.G95** | CD982561 |
| 9 | M | Crimea | *57 kb deletion* | CG004969 | *c.1000del, p.T334Pfs*65* | n/a |
| 10 | M | Krasnodar Krai | *57 kb deletion* | CG004969 | *c.18_21del, p.T7Ffs*7* | CD982561 |
| 11 | F | Belarus | *c.140+2dup* | n/a | *c.140+2dup* | n/a |
| 12 | F | Republic of Kabardino-Balkaria | *57 kb deletion* | CG004969 | *57 kb deletion* | CG004969 |
| 13 | M | Moscow Oblast | *c.433C>T, p.Q145** | CM195019 | *c.433C>T, p.Q145** | CM195019 |
| 14 | M | Orenburg Oblast | *c.433C>T, p.Q145** | CM195019 | *c.681+1G>A* | CS993011 |
| 15 | F | Tatarstan | *57 kb deletion* | CG004969 | *c.785G>A, p.W262** | n/a |
| 16 | F | n/a | *57 kb deletion* | CG004969 | *57 kb deletion* | CG004969 |
| 17 | F | Tatarstan | *c.785G>A, p.W262** | n/a | *c.785G>A, p.W262** | n/a |
| 18 | M | Komi | *57 kb deletion* | CG004969 | *c.433C>T, p.Q145** | CM195019 |
| 19 | M | Bashkortostan | *57 kb deletion* | CG004969 | *c.785G>A, p.W262** | n/a |
| 20 | M | Moscow Oblast | *57 kb deletion* | CG004969 | *c.785G>A, p.W262** | n/a |
| 21 | M | Bashkortostan? | *c.627C>A, p.S209R* | n/a | *c.627C>A, p.S209R* | n/a |
| 22 | F | Chechnya | *c.518A>G, p.Y173C* | CM1110322 | *c.518A>G, p.Y173C* | CM1110322 |
| 23 | M | Novosibirsk Oblast | *57 kb deletion* | CG004969 | *c.505G>T, p.G169C* | n/a |
| 24 | F | Chechnya | *c.518A>G, p.Y173C* | CM1110322 | *c.518A>G, p.Y173C* | CM1110322 |
| 25 | M | Mordovia | *c.413G>A, p.W138** | n/a | *c.433C>T, p.Q145** | CM195019 |
| 26 | M | Saint-Petersburg | *57 kb deletion* | CG004969 | *c.323del, p.Q108Rfs*10* | CD172137 |
| 27 | M | Moscow Oblast | *57 kb deletion* | CG004969 | *c.518A>G, p.Y173C* | CM1110322 |
| 28 | F | Kostroma | *57 kb deletion* | CG004969 | *57 kb deletion* | CG004969 |
| 29 | M | Chechnya | *c.518A>G, p.Y173C* | CM1110322 | *c.518A>G, p.Y173C* | CM1110322 |
| 30 | M | Republic of Kabardino-Balkaria | *c.1015G>A, p.G339R* | CM980461 | *c.681G>A, p.E227E* | CS099126 |
| 31 | M | Novosibirsk Oblast | *57 kb deletion* | CG004969 | *57 kb deletion* | CG004969 |
| 32 | M | Ingushetia | *c.518A>G, p.Y173C* | CM1110322 | *c.518A>G, p.Y173C* | CM1110322 |
| 33 | M | Dagestan | *g.(?_3550706)_(3552123_?)del* | CG994863 | *g.(?_3550706)_(3552123_?)del* | CG994863 |
| 34 | F | Karachay-Cherkessia | *c.1015G>A, p.G339R* | CM980461 | *c.1015G>A, p.G339R* | CM980461 |
| 35 | M | Ukraine | *g.(?_ 3558266)_( 3558736_?)del* | n/a | *g.(?_3558266)_( 3558736_?)del* | n/a |
| 36 | F | Saint-Petersburg | *с.451A>G, p.R151G* | CM1110322 | *с.451A>G, p.R151G* | CM1110322 |
| 37 | F | Ukraine | *c.18_21del, p.T7Ffs*7* | CD982561 | *с.613G>A, p.D205K* | CM980461 |
| 38 | M | Ukraine | *с.699_700del, p.S234Lfs*61* | CD031495 | *с.699_700del, p.S234Lfs*61* | CD031495 |
| 39 | M | Omsk Oblast | *c.198_218del p.(Ile67_Pro73del)* | n/a | *g.(?_3558266)_(3565849_?)del* | n/a |
| 40 | F | Smolensk Oblast | *c.433C>T, p.Q145** | CM195019 | *g.(?_3558266)_(3565849_?)del* | n/a |

**Supplementary 5**

**Table 2.** Clinical features of patients

| **N** | **Sex** | **Age of onset, months** | **Vomiting** | **Hepatomegaly** | **Splenomegaly** | **Muscle weakness** | **Polydipsia / Polyuria** | **Failure to thrive** | **Developmental delay** | **Сhronic kidney disease at the time of diagnosis** | **Fanconi syndrome (secondary)** | **Eye injury (keratitis)** | **Rickets-like changes** |
| --- | --- | --- | --- | --- | --- | --- | --- | --- | --- | --- | --- | --- | --- |
| 1 | F | 6 | yes | no | no | yes | yes | yes | yes | 1 | yes | yes | no |
| 2 | F | 3,5 | yes | yes | no | yes | yes | yes | yes | 2 | yes | yes | no |
| 3 | F | 3 | no | yes | yes | yes | yes | yes | no | 1 | yes | yes | no |
| 4 | F | 6 | yes | yes | no | yes | yes | yes | no | 2 | yes | yes | no |
| 5 | F | 4 | yes | no | no | yes | yes | yes | yes | 1 | yes | yes | yes |
| 6 | M | 5 | no | yes | no | yes | yes | yes | no | 1 | yes | yes | no |
| 7 | M | 7 | no | yes | no | yes | yes | yes | yes | 1 | yes | yes | yes |
| 8 | M | 12 | yes | no | no | yes | yes | yes | yes | 1 | yes | yes | yes |
| 9 | M | 18 | yes | no | no | no | yes | no | no | 5 | yes | yes | yes |
| 10 | M | 12 | yes | n/a | n/a | yes | yes | yes | yes | 5 | yes | yes | no |
| 11 | F | 144 | no | n/a | n/a | yes | no | yes | no | no | yes | yes | no |
| 12 | F | 6 | n/a | yes | yes | no | yes | yes | no | 1 | yes | yes | yes |
| 13 | M | 4 | yes | n/a | n/a | yes | yes | yes | yes | no | yes | yes | no |
| 14 | M | 18 | no | yes | no | yes | yes | yes | no | 1 | yes | yes | yes |
| 15 | F | 15 | yes | no | no | no | yes | yes | no | 2 | yes | yes | yes |
| 16 | F | 132 | n/a | n/a | n/a | no | yes | yes | no | no | yes | yes | no |
| 17 | F | 6 | no | no | no | yes | yes | yes | yes | 3 | yes | yes | no |
| 18 | M | 3 | no | yes | no | yes | yes | yes | no | 1 | yes | yes | no |
| 19 | M | 6 | yes | yes | no | yes | yes | yes | yes | 3 | yes | yes | yes |
| 20 | M | 10 | no | yes | no | no | no | no | no | no | yes | no | no |
| 21 | M | 18 | no | no | no | yes | yes | yes | no | 5 | yes | yes | yes |
| 22 | F | 16 | yes | yes | no | yes | yes | yes | yes | 1 | yes | yes | n/a |
| 23 | M | 12 | no | no | no | no | n/a | yes | no | 5 | yes | yes | yes |
| 24 | F | 18 | yes | yes | no | yes | yes | yes | yes | 4 | yes | yes | yes |
| 25 | M | 7 | no | no | no | yes | yes | yes | no | no | yes | yes | yes |
| 26 | M | 2 | yes | yes | yes | yes | yes | yes | yes | 5 | yes | yes | yes |
| 27 | M | 4 | нет | no | no | yes | no | yes | no | 1 | yes | yes | no |
| 28 | F | 7 | н/д | n/a | n/a | yes | yes | yes | yes | n/a | yes | yes | no |
| 29 | M | 15 | no | yes | yes | yes | yes | yes | no | 1 | yes | no | yes |
| 30 | M | 8 | yes | yes | yes | yes | yes | no | no | 1 | yes | yes | yes |
| 31 | M | 6 | yes | yes | yes | yes | yes | yes | no | 2 | yes | yes | yes |
| 32 | M | 7 | yes | yes | yes | yes | yes | yes | no | 1 | yes | yes | yes |
| 33 | M | 7 | no | no | no | yes | yes | yes | n/a | 3 | yes | yes | yes |
| 34 | F | 12 | yes | no | no | yes | yes | yes | no | 4 | yes | yes | yes |
| 35 | M | n/a | n/a | n/a | n/a | n/a | n/a | n/a | n/a | n/a | n/a | n/a | n/a |
| 36 | F | n/a | n/a | n/a | n/a | n/a | n/a | n/a | n/a | n/a | n/a | n/a | n/a |
| 37 | F | n/a | n/a | n/a | n/a | n/a | n/a | n/a | n/a | n/a | n/a | n/a | n/a |
| 38 | M | n/a | n/a | n/a | n/a | n/a | n/a | n/a | n/a | n/a | n/a | n/a | n/a |
| 39 | M | 72 | no | no | no | no | no | no | no | 5 | yes | yes | no |
| 40 | F | 18 | no | no | no | no | yes | yes | no | 4 | yes | yes | no |

**Supplementary 6**

**Table 3.** Allele frequencies of microsatellite markers on chromosomes with the c.1015G>A mutation (D) and on chromosomes without the mutation (N).

| Marker | **D17S831** | | **D17S1798** | | **D17S829 (CTNS)** | | **D17S1828** | | **D17S1876** | |
| --- | --- | --- | --- | --- | --- | --- | --- | --- | --- | --- |
|  | D | N | D | N | D | N | D | N | D | N |
| Allele | Number of alleles/ frequencies (%) | | | | | | | | | |
| 1 | **7/87.5** | 3/8.3 | **5/62.5** | 17/47.2 |  | 10/27.8 |  | 1/2.7 |  | 5/13.9 |
| 2 |  |  | 2/25 | 16/44.4 |  | 1/2.7 |  | 9/25.0 |  | 8/22.2 |
| 3 |  | 9/25.0 | 1/12.5 | 1/2.7 | **8/100** | 12/33.3 | **8/100** | 12/33.3 |  | 4/11.1 |
| 4 |  | 13/36.1 |  | 1/2.7 |  | 13/36.1 |  | 3/8.3 |  |  |
| 5 |  | 1/2.7 |  | 1/2.7 |  |  |  | 8/22.2 |  | 2/5.6 |
| 6 |  | 3/8.3 |  |  |  |  |  | 1/2.7 |  | 6/16.7 |
| 7 |  | 3/8.3 |  |  |  |  |  |  |  | 6/16.7 |
| 8 | 1/12.5 | 2/5.6 |  |  |  |  |  | 1/2.7 |  | 3/8.3 |
| 9 |  | 1/2.7 |  |  |  |  |  | 1/2.7 | **7/87.5** | 2/5.6 |
| 10 |  | 1/2.7 |  |  |  |  |  |  | 1/12.5 |  |
| Number of examined chromosomes | 8 | 36 | 8 | 36 | 8 | 36 | 8 | 36 | 8 | 36 |

The frequencies of alleles that showed maximal linkage disequilibrium with the c.1015G>A mutation are underlined.

**Supplementary 7**

**Table 4.** Allele frequencies of microsatellite markers on chromosomes with the c.518A>G mutation (D) and on chromosomes without the mutation (N).

| Marker | **D17S831** | | **D17S1798** | | **D17S829 (CTNS)** | | **D17S1828** | | **D17S1876** | |
| --- | --- | --- | --- | --- | --- | --- | --- | --- | --- | --- |
|  | D | N | D | N | D | N | D | N | D | N |
| Allele | Number of alleles/ frequencies (%) | | | | | | | | | |
| 1 | 1/12.5 | 5/14.7 | 4/50.0 | 16/50.0 | **8/100** | 10/29.4 |  | 1/2.9 | 1/12.5 | 9/26.5 |
| 2 |  | 1/2.9 | 3/37.5 | 13/40.6 |  | 1/2.9 | 1/12.5 | 3/8.8 |  | 1/2.9 |
| 3 | 2/25.0 | 9/26.5 | 1/12.5 |  |  | 1/2.9 |  | 5/14.7 |  |  |
| 4 | 2/25.0 | 5/14.7 |  |  |  | 19/55.9 |  | 4/11.8 |  |  |
| 5 |  | 1/2.9 |  | 1/3.1 |  |  | **5/62.5** | 10/29.4 |  | 2/5.9 |
| 6 | **2/25.0** | 3/8.8 |  |  |  | 1/2.9 |  | 2/5.9 |  | 4/11.8 |
| 7 |  | 5/14.7 |  |  |  |  |  | 1/2.9 | **7/87.5** | 3/8.8 |
| 8 |  | 3/8.8 |  |  |  | 1/2.9 | 2/25.0 | 2/5.9 |  | 6/17.6 |
| 9 | 1/12.5 | 1/2.9 |  | 2/6.3 |  |  |  | 4/11.8 |  | 4/11.8 |
| 10 |  |  |  |  |  | 1/2.9 |  |  |  | 5/14.7 |
| 11 |  | 1/2.9 |  |  |  |  |  | 2/5.9 |  |  |
| Number of examined chromosomes | 8 | 34 | 8 | 32 | 8 | 34 | 8 | 34 | 8 | 34 |

The frequencies of alleles that showed maximal linkage disequilibrium with the c.518A>G mutation are underlined.
